# Multi-cell-component cartilage organoids simulate intercellular microstress, hypoxic microenvironment, and chondrocyte–endothelial crosstalk *in vitro*

**DOI:** 10.3389/fbioe.2026.1852010

**Published:** 2026-07-02

**Authors:** Zhengchao Wang, Pengfei Zhu, Hongmei Li, Qingsong Zhang, Jiangxia Cheng, Yu Cai

**Affiliations:** 1 Department of Sports Medicine, Wuhan Fourth Hospital, Wuhan, China; 2 Hubei Provincial Sports Medicine Center, Wuhan, China; 3 Hubei Key Laboratory of Sports Injury and Precision Therapy, Wuhan Fourth Hospital, Wuhan, China; 4 Hubei Provincial Clinical Research Center for Orthopaedics, Wuhan, China; 5 Department of Cardiovascular, Wuhan Fourth Hospital, Wuhan, China; 6 Zibo First Hospital, Zibo Prevention and Treatment Hospital for Occupation Diseases, Zibo, China; 7 Department of Anesthesiology, Wuhan Fourth Hospital, Wuhan, China; 8 Department of Rehabilitation, Wuhan Fourth Hospital, Wuhan, China

**Keywords:** chondrocyte, vessel invasion, endothelial cell, organoid, osteoarthritis

## Abstract

**Introduction:**

In cartilage-related studies, the existing *in vitro* cartilage models often fail to simulate the complex pathophysiology involving a hypoxic microenvironment, abnormal intercellular mechanical stress (microstress), and pathological crosstalk between chondrocytes and vascular endothelial cells. he aim of this study was to develop and validate a novel, simplified multi-cell-component cartilage organoid model capable of mimicking these three key OA-related features *in vitro*, using readily available cell lines.

**Methods:**

Organoids were constructed using SW1353 chondrocytes and HMEC-1 endothelial cell lines within low-adhesion grid microchambers. A monosodium iodoacetate (MIA)-induced OA model was developed. Histological and immunofluorescence analyses of the organoids were conducted to examine markers of matrix metabolism, cytoskeletal and intercellular microstress, hypoxic microenvironment, and chondrocyte -endothelial crosstalk. The organoid features were compared with those of standard 2D cell cultures and *in vivo* cartilages.

**Results:**

The multi-cell-component organoids successfully recapitulated critical *in vivo* features. They exhibited MIA-induced F-actin reorganization and PIEZO1 upregulation, developed a hypoxic core with elevated hypoxia-inducible factor 1-alpha (HIF-1α) levels, and formed an endothelial shell that invasively disrupted the chondrocyte core upon MIA treatment. This disruption was accompanied by the activation of vascular endothelial growth factor -NOTCH receptor 1 -delta-like ligand 4 (VEGF -NOTCH1 -DLL4) signaling, mirroring the pathological chondrocyte -endothelial crosstalk. Single-cell-component organoids simulated microstress-related changes but not hypoxia or chondrocyte -endothelial crosstalk.

**Discussion:**

The multi-cell-component cartilage organoids constructed from tumor-derived cell lines using low-adhesion grid microchambers partially simulated intercellular microstress, hypoxic microenvironment, and chondrocyte -endothelial crosstalk, which was similar to that observed in cartilages in vivo. These organoids provide a new strategy for conducting cartilage-related studies *in vitro*.

## Introduction

Chondrocytes are the only resident cells in cartilage. However, previous studies have shown that cartilage pathology involves multiple factors and cells. Most of the cartilage lesions are initiated by, and result from, abnormal stress conditions (Brylka et al., 2024). Abnormal changes in the microstructure of a cartilage, including the cytoskeleton and extracellular matrix, contribute to abnormal intercellular microstress (Gonzalez-Nolde et al., 2024). This leads to the activation of stress-sensitive ion channels like PIEZO1, further changing the structures of chondrocytes and cartilage, which enhances cartilage damage (Brylka et al., 2024; Jiang et al., 2023; Li et al., 2022; Xiao et al., 2025). Alternatively, cartilage lesions are initiated by, and result from, abnormal crosstalk between the cartilage and vessel endothelium. During cartilage lesion development and degeneration, abnormal crosstalk between the chondrocytes and vessel endothelium leads to vascular invasion of the cartilage (Liu et al., 2025; Wang et al., 2025b). The vascular invasion results in collapse of the cartilage microenvironment and promotes the development of cartilage lesions (Qin et al., 2019; Wang et al., 2025a; Zhang et al., 2023). Unfortunately, these two types of progressions are difficult to be simulated in simple cell cultures *in vitro*. Therefore, organoids and 3D cell cultures could be a feasible solution for this problem.

In recent decades, technologies focusing on organoids and 3D cell cultures have developed rapidly. Significant breakthroughs have been achieved in the development of organoids derived from epithelial tissues such as small intestine, liver, kidney, lung, and retina (Zeng et al., 2023). However, regarding cartilage organoids derived from mesenchymal tissues, some limitations remain. Previous studies have mainly focused on stem cells, such as embryonic stem cells (ESCs), induced pluripotent stem cells (iPSCs), mesenchymal stem cells (MSCs), and adult stem cells (ASCs), with self-renewal and partial physiological properties (Abe et al., 2023; Abraham et al., 2022; Jiang et al., 2021; Lin et al., 2023). However, the highly technical demands and critical culture conditions make large-scale application of stem cells difficult. Therefore, a simple and convenient method of generating organoids from cartilage needs to be developed.

Cell lines derived from tumor tissues *in vitro* have been widely applied in disease-related studies. These cell lines not only simulate the cellular changes *in vivo* but are also easy to be cultured and treated. In a previous study, we used SW1353, a cell line derived from chondromas, to generate organoids for cartilage microstructure analysis; the findings showed that 3D-cultured cell lines could be used to generate organoids within low-adhesion grid microchambers for *in vitro* studies (Wang et al., 2025a). In this study, we aimed to construct multi-cell organoids using different cell lines and low-adhesion grid microchambers to develop an appropriate cartilage organoid model *in vitro*, which could react to abnormal stress changes and chondrocyte–endothelial crosstalk. We used monosodium iodoacetate (MIA), a common reagent used to build *in vitro* and *in vivo* osteoarthritis (OA) models, to analyze the applications of organoids in cartilage-related diseases.

## Methods

### Cell culture and organoid construction

SW1353 cells were cultured in high-glucose Dulbecco’s modified Eagle medium (DMEM) supplemented with 10% fetal calf serum and 1% penicillin–streptomycin at a density of 1 × 10^5^ cells/mL (1 × 10^5^ cells/well). HMEC-1 cells were cultured in endothelial cell-specific culture medium (Procell, Wuhan, China) with 10% fetal calf serum and 1% penicillin–streptomycin at a density of 1 × 10^5^ cells/mL (1 × 10^5^ cells/well). Cells were incubated in a humidified atmosphere of 5% CO_2_ at 37 °C. The culture medium was refreshed every 2–3 days, and the cells were passaged using a 0.25% trypsin–EDTA solution when 80%–90% confluence was attained.

**FIGURE 1:**
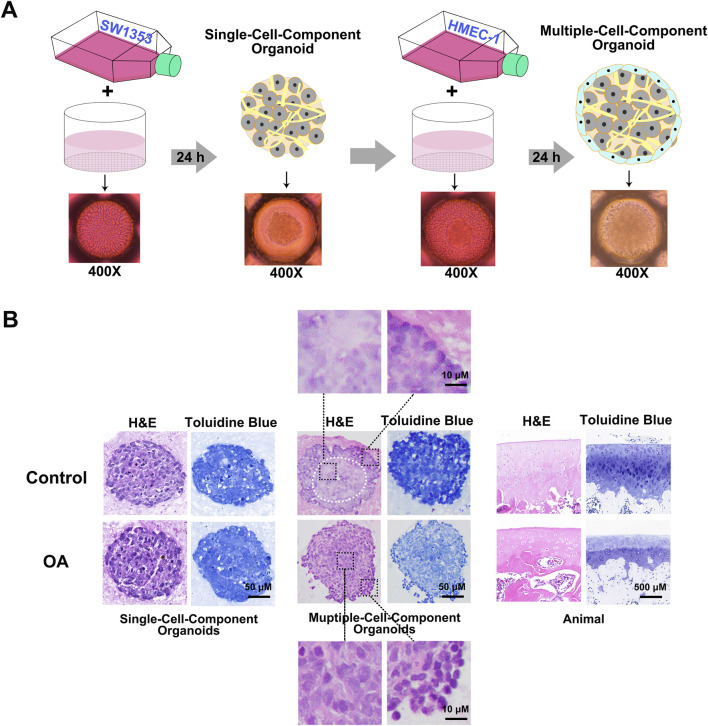
**(A)** Scheme of organoid construction using low-adhesion grid microchambers. **(B)** Histological features of hematoxylin and eosin (H&E)- and toluidine blue-stained single- and multi-cell-component organoids *in vitro* and cartilage *in vivo*.

For organoid construction, as shown in Figure 1A, SW1353 cells were digested with 0.25% trypsin–EDTA solution to obtain a cell suspension (1 × 10^6^ cells/mL). The low-adhesion grid microchambers (CSwell 600; Jiyan Biology, Suzhou, China), designed for high-throughput 3D cell cultures, were pretreated according to the manufacturer’s instructions. One milliliter of SW1353 cell suspension was added per well to construct chondrocyte organoids comprising 1,000–2,000 cells. After 24 h incubation, the single-cell organoids were constructed completed (Figure 1A). Based on the single-cell organoids, HMEC-1 cells were digested with 0.25% trypsin–EDTA solution to obtain a cell suspension (1 × 10^6^ cells/mL) and added to the same chamber. After 24 h incubation, a shell constructed by HMEC-1 cells could be observed outside the SW1353 core and the multiple-cell organoids were constructed completed (Figure 1A). Organoid development was confirmed by observation using an inverted microscope (Leica).

### Induction of monosodium iodoacetate (MIA)-induced inflammation

MIA was purchased from Aladdin (Shanghai, China), dissolved in normal saline, and stored according to the manufacturer’s instructions. Wells loaded with organoids were incubated with or without MIA for 24 h. Subsequently, the organoids were embedded in SE-GEL (Jiyan Biology) according to the manufacturer’s instructions.

### Animals

Male Sprague–Dawley rats (160–200 g) were purchased from the Experimental Animal Center, Tongji Medical College, Huazhong University of Science and Technology, China. Animals were fed a normal diet and housed under a 12-h light/dark cycle under controlled humidity and temperature conditions (25 °C). Left knee joint OA was induced by intraarticular injection of 3 mg/50 μL MIA or 50 μL normal saline under 1% pentobarbital anesthesia (80 mg/kg intraperitoneally [i.p.]) using a 27-gauge needle inserted through the patellar tendon. After 3 weeks, all animals were euthanized using an overdose of 10% urethane. The knee joints were dissected for histopathological analysis. All experimental animals were maintained in accordance with the Guide for the Care and Use of Laboratory Animals of the National Institutes of Health, and the protocols were approved by the Ethics Committee of Wuhan Fourth Hospital.

### Histological analysis

Embedded organoids were fixed in 4% paraformaldehyde for 24 h. Rat knee joint samples were fixed in 4% paraformaldehyde for 24 h and then decalcified in 10% EDTA for 4 weeks. After paraffin embedding, 4-μm-thick sections were obtained for histological analysis. Hematoxylin and eosin (H&E) and toluidine blue staining were performed.

### Immunofluorescence (IF) analysis

Histological slides were prepared as described in the previous paragraph. Primary antibodies were diluted according to the manufacturer’s instructions. The following primary antibodies were used: collagen II (1:500; rabbit; Proteintech); collagen X (1:500; rabbit; Proteintech); aggrecan (1:500; rabbit; Proteintech); matrix metalloproteinase-3 (MMP-3; 1:500; rabbit; Proteintech); F-actin (phalloidin; 1:200; Proteintech); hypoxia-inducible factor 1-alpha (HIF-1α; 1:500; mouse; Proteintech); vascular endothelial growth factor (VEGF; 1:300; rabbit; Proteintech); NOTCH receptor 1 (NOTCH1; 1:200; rabbit; Proteintech); delta-like ligand 4 (DLL4; 1:200; rabbit; Proteintech); endomucin (EMCN; 1:500; rabbit; Proteintech); CD31 (1:500; mouse; Proteintech). After deparaffinization and rehydration, the samples were incubated overnight with diluted primary antibodies at 4 °C. Fluorescein-labeled secondary antibodies were added to the samples, and the samples were incubated for 1 h at room temperature. Nuclei were stained with 4′,6-diamidino-2-phenylindole (DAPI), and the slides were scanned using an LSM 710 confocal microscope (Zeiss, Oberkochen, Germany) with an EC-Plan-Neofluar 40×/1.3 oil immersion objective. Quantitative data were obtained from the fluorescence intensity measurements using the ZEN 2009 software (Zeiss).

### Statistical analyses

Statistical analyses were performed using IBM SPSS Statistics for Windows, version 21.0 (IBM Corp., Armonk, NY, USA). The normality of data distribution was tested using a Q‒Q plot. The data were analyzed using repeated-measures analysis of variance (ANOVA). A *post hoc* test was performed with Bonferroni correction. A p-value < 0.05 was considered statistically significant.

## Results

### Construction of single- and multi-cell-component organoids

The SW1353 cells were incubated in low-adhesion CSwell 600 grid microchambers (Figure 1A). After 24-h incubation, the single-cell-component organoids were established. Subsequently, HMEC-1 cells were added to the CSwell 600 microchambers to build an endothelial shell around the chondrocytes. After 24-h incubation, the HMEC-1 cells gathered into a mass, and the multiple-cell-component organoids were established. The organoids were then incubated with or without MIA for 24 h for inflammation induction.

### Histological features of single- and multi-cell-component organoids

The histological features of the organoids were detected by H&E and toluidine blue staining. After H&E staining, chondrocytes in the organoids appeared light red, and the morphology of the cells and extracellular matrix was similar to that of cartilages *in vivo* (Figure 1B). A boundary could be observed in multi-cell-component organoids between the regions containing chondrocytes and endothelial shells, similar to that between the cartilage and subchondral bone *in vivo*. After MIA induction, no significant histological changes could be observed in single-cell-component organoids compared with that in the control. However, in multi-cell-component organoids, the chondrocyte–endothelial boundary was disrupted and indistinct, which resembled cartilage degeneration *in vivo*. After toluidine blue staining, no distinct boundary could be observed between chondrocytes and endothelial cells in multi-cell-component organoids. However, after OA induction, toluidine blue staining intensity decreased in the multi-cell-component organoids, which indicates cartilage matrix depletion.

### Matrix metabolism biomarkers in single- and multi-cell-component organoids

**FIGURE 2:**
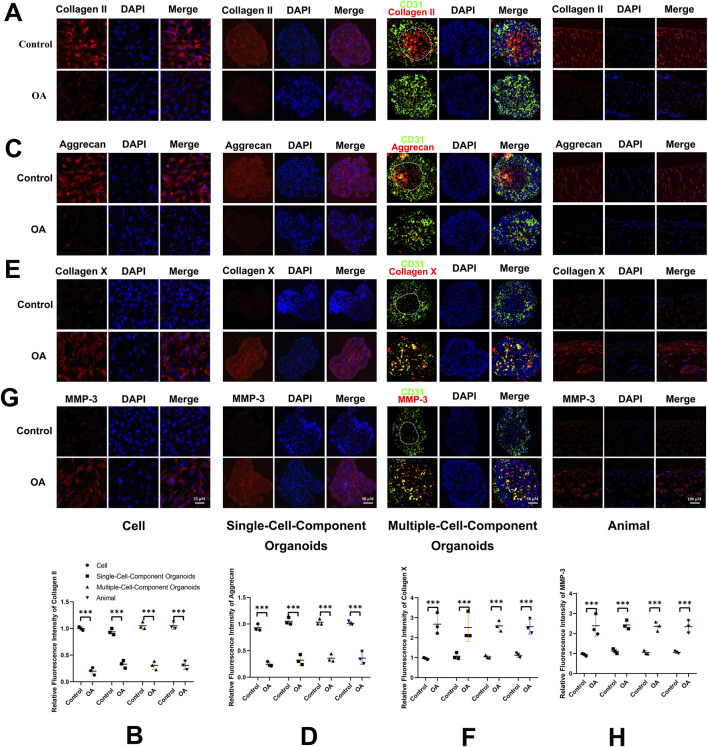
**(A-H)** Cartilage matrix and anabolism could be simulated by single- and multiple-cell component organoids. Cartilage matrix anabolism- and catabolism-related biomarkers in normal and osteoarthritis (OA)-associated chondrocytes in cell cultures, single- and multi-cell-component organoids, and cartilage in vivo, detected using immunofluorescence (IF) analysis. N=3 in all groups. The statistical graph is plotted based on the relative fluorescence intensity. ***p < 0.001.

The cartilage matrix anabolism- and catabolism-related biomarkers were detected using IF analysis. Collagen II and aggrecan levels decreased, whereas collagen X and MMP3 levels increased after MIA induction compared with the preinduction levels in the two organoids, which were similar to that observed in chondrocytes and cell cultures *in vitro* and cartilages *in vivo* (Figure 2). Moreover, in multi-cell-component organoids, a distinct boundary was observed between CD31-positive endothelial cells and chondrocyte marker-positive regions. After OA induction, the organoid structure collapsed into a disorganized mass, and the boundary became indistinct.

### Cytoskeleton formation and intercellular microstress in single- and multi-cell-component organoids

**FIGURE 3:**
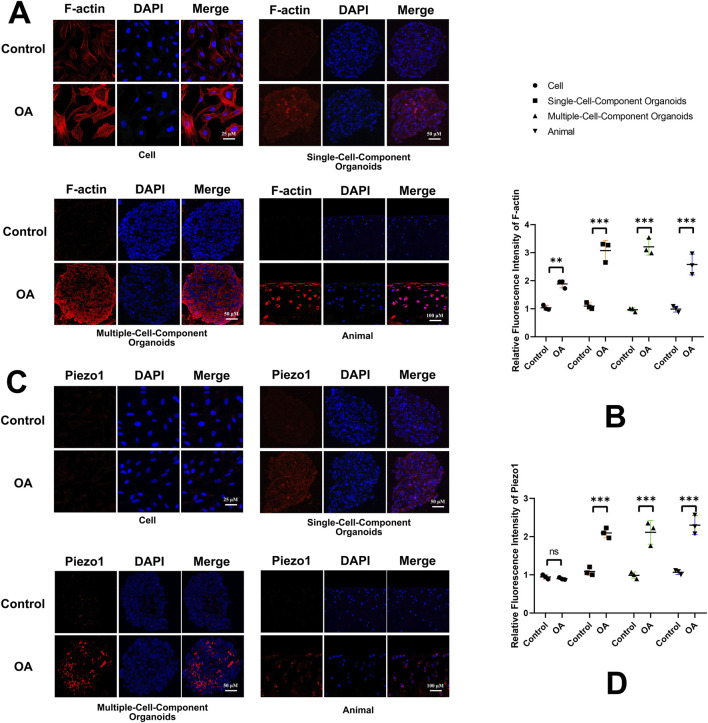
Cytoskeleton formation and intercellular microstress could be simulated by single- and multiple-cell component organoids. **(A,B)** IF analysis of F-actin for investigation of cytoskeleton formation in normal and OA chondrocytes in cell cultures, single- and multi-cell-component organoids, and cartilage *in vivo*. **(C,D)** IF analysis of stress-sensitive ion channel PIEZO1 in normal and OA chondrocytes in cell cultures, single- and multi-cell-component organoids, and cartilage *in vivo*. ns, no significant difference; N = 3 in all groups. The statistical graph is plotted based on the relative fluorescence intensity. **p < 0.01; ***p < 0.001.

Cytoskeleton formation was detected using F-actin IF analysis. F-actin formation increased after MIA induction compared with that before induction in the two organoids, which was similar to that observed in chondrocytes *in vitro* and cartilages *in vivo* (Figures 3A,B). However, no distinct linear structure was observed in the organoids, which was similar to that in cartilages *in vivo* and unlike that in chondrocytes *in vitro*. The intercellular microstress was detected using IF analysis of PIEZO1, a stress-sensitive ion channel. The PIEZO1 levels were similar before and after MIA induction of chondrocytes *in vitro* (Figures 3C,D). The PIEZO1 levels were significantly upregulated after MIA induction compared with that before the induction in the two organoids, which was similar to that observed in cartilages *in vivo*. These results indicate that the single- and multi-cell component organoids simulated the intercellular microstress changes observed after cytoskeleton formation in OA models.

### Hypoxic microenvironment in single- and multi-cell-component organoids

**FIGURE 4:**
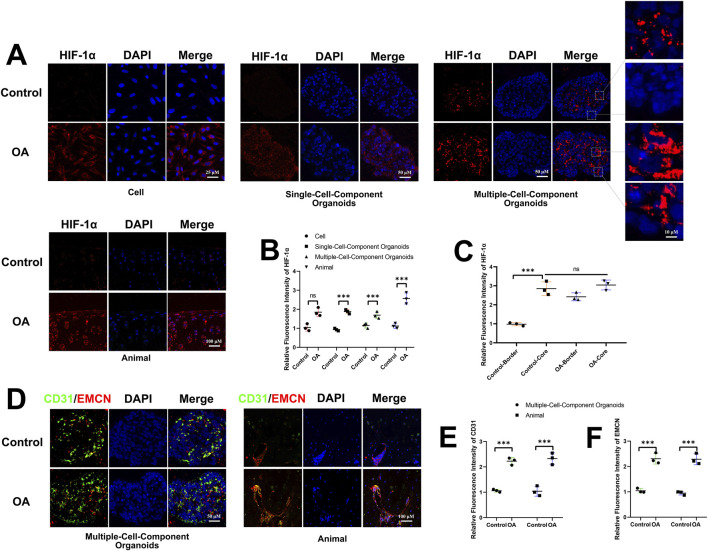
Hypoxic microenvironment and vascular invasion could be simulated by multiple-cell component organoids. **(A–C)** IF analysis of the hypoxic marker hypoxia-inducible factor 1-alpha (HIF-1α) in normal and OA chondrocytes in cell cultures, single- and multi-cell-component organoids, and cartilage *in vivo*. **(D–F)** IF analysis of the endothelial markers CD31 and endomucin (EMCN) in normal and OA cartilage chondrocytes in multi-cell-component organoids and cartilage *in vivo*. ns, no significant difference; N = 3 in all groups. The statistical graph is plotted based on the relative fluorescence intensity. ***p < 0.001.

To detect the hypoxic microenvironment, IF staining was performed to analyze the levels of HIF-1α, an oxygen-sensitive and inflammation-related transcription factor. The HIF-1α levels were low in chondrocytes *in vitro* and single-cell-component organoids compared with that in the control (Figures 4A,B). The core regions of multi-cell-component organoids exhibited high levels of HIF-1α compared with that in the control, which indicates the presence of a hypoxic microenvironment in multi-cell-component organoids similar to that in cartilages *in vivo* (Figures 4A,C). After MIA induction, the HIF-1α levels significantly increased compared with the preinduction levels in the chondrocytes, two kinds of organoids, and cartilage. These results indicate that the multi-cell-component organoids partially simulated the hypoxic microenvironment of cartilage.

### Chondrocyte–endothelial crosstalk in multi-cell-component organoids

The chondrocyte–endothelial crosstalk was analyzed using multi-cell-component organoids and cartilages *in vivo*. The endothelial morphology was examined using IF analysis of CD31 and EMCN. A distinct endothelial shell was observed around the chondrocyte region in multi-cell-component organoids, similar to that observed between the cartilage and subchondral bone *in vivo* (Figures 4D–F). After MIA induction, the endothelial shell collapsed into a disorganized mass, with the endothelial region invading the chondrocyte region; this process was similar to the vascular invasion observed in OA-affected cartilage and subchondral bone.

**FIGURE 5:**
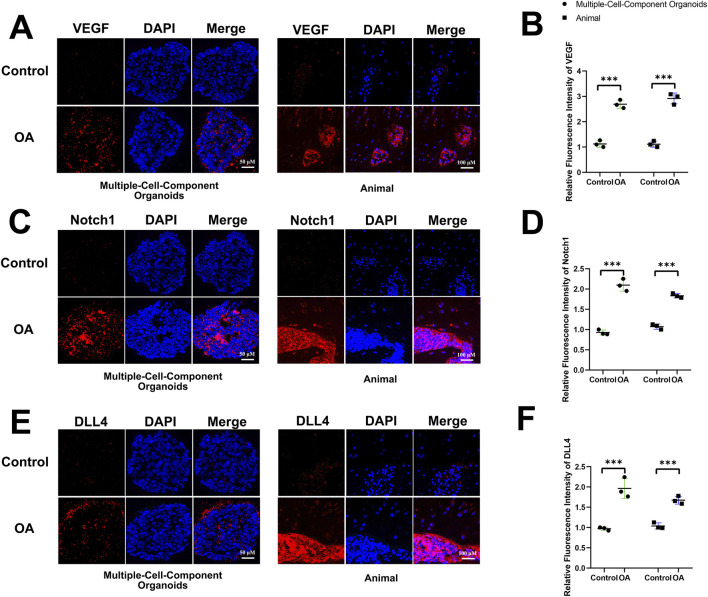
**(A-F)** Chondrocyte -endothelial crosstalk could be simulated by multiple-cell component organoids. IF analysis of vascular endothelial growth factor -NOTCH receptor 1 -delta-like ligand 4 (VEGF -NOTCH1 -DLL4)-mediated chondrocyte -endothelial crosstalk in normal and OA cartilage chondrocytes in multi-cell-component organoids and cartilage *in vivo*. N=3 in all groups, and the statistical graph is plotted based on the relative fluorescence intensity. N=3 in all groups. The statistical graph is plotted based on the relative fluorescence intensity. ***p < 0.001.

Subsequently, the VEGF, NOTCH1, and DLL4 levels were detected by IF analysis. The VEGF, NOTCH1, and DLL4 levels increased significantly after MIA induction compared with the preinduction levels in organoids *in vitro* and cartilages *in vivo*, particularly at the boundary region between the endothelial shell and chondrocytes (Figure 5). This phenomenon was also observed in cartilage and subchondral bone *in vivo*. These results indicate that the multi-cell-component organoids mimicked the chondrocyte–endothelial crosstalk observed between cartilage and subchondral bone.

## Discussion

In this study, we applied a simple method and two common tumor-derived cell lines to generate organoids for cartilage-related research. We found that the multi-cell-component organoids generated using SW1353 + HMEC-1 cells in low-adhesion CSwell 600 grid microchambers simulated the intercellular microstress changes that occur during cytoskeleton formation, hypoxic microenvironment, and chondrocyte–endothelial crosstalk observed in cartilage *in vivo*. Moreover, the single-cell-component organoids constructed using SW1353 cells simulated the intercellular microstress changes. Overall, cartilage-related studies can be conducted using *in vitro* organoids to mitigate the limitations of traditional cell cultures.

Previous studies have shown that hypoxic environment is important for cartilage homeostasis (Zhang et al., 2023). Notably, an oxygen-sensitive transcription factor HIF-1α regulates the metabolism, morphogenesis, and survival of chondrocytes (Hu et al., 2020; Sanz-Ramos et al., 2013; Taheem et al., 2020; Zhu et al., 2020). The HIF-1α levels are upregulated by hypoxia and inflammation (Hu et al., 2020; Zhu et al., 2020). However, *in vivo* studies have shown that HIF-1α levels are downregulated in OA mouse models (Zhang et al., 2023). Previously, we reported that the reasons for this contradiction in results were a damaged subchondral bone barrier and vascular invasion (Wang et al., 2025b). During the early stages of OA, a stable hypoxic microenvironment is maintained under conditions of a relatively intact subchondral bone barrier and slight vascular invasion (Wang et al., 2025b). In this condition, the HIF-1α levels are only affected by inflammation (Wang et al., 2025b). With OA progression, the breakdown of the subchondral bone barrier and vascular invasion lead to a collapse of the hypoxic microenvironment in the cartilage, causing a decrease in HIF-1α levels because of the increase in oxygen concentration (Wang et al., 2025b). In our previous studies, we applied different oxygen concentrations in cell cultures to mimic the hypoxic microenvironment (Wang et al., 2025a; Wang et al., 2025b). In this study, the multi-cell-component organoids, comprising a chondrocyte core surrounded by endothelial shells. The organoids exhibited a hypoxic core with high HIF-1α levels, thereby mimicking the conditions inside cartilage *in vivo*.

The cytoskeleton in chondrocytes is another factor associated with cartilage degeneration. In normal chondrocytes, the cytoskeleton mainly comprises dissociative G-actin with small amounts of F-actin (Rzepski et al., 2025). During OA progression, G-actin polymerizes into linear F-actin fibers, resulting in the increase in stiffness of chondrocytes (Gonzalez-Nolde et al., 2024; Parreno et al., 2017; Plaas et al., 2011; Rzepski et al., 2025). These changes in chondrocyte skeletal structure and microstructure result in abnormal intercellular microstress and altered chemical signaling (Garcia-Castorena et al., 2025; Gonzalez-Nolde et al., 2024). Abnormal stress activates PIEZO1 in chondrocytes, triggering a series of stress-sensitive signaling pathways that accelerate OA degeneration (Brylka et al., 2024; Xiao, 2024). However, this phenomenon is difficult to simulate *in vitro*. Chondrocyte dedifferentiation was markedly upregulated *in vitro*. Although inflammation affects the cytoskeleton, F-actin is the predominant form of actin in chondrocytes. Moreover, intercellular microstress conditions cannot be simulated in cell cultures. Therefore, *in vivo* studies are ideal for examining stress-sensitive proteins. A similar process can only be simulated *in vitro* via chemical activation using agonists such as Yoda1 (Garcia-Castorena et al., 2025; Wang et al., 2025). In this study, we developed 3D chondrocyte organoids to inhibit dedifferentiation and simulate intercellular microstress. Although tumor-derived cell lines were used, the cells exhibited a response to the inflammatory conditions (Wang et al., 2025a). Organoids developed from cell lines in 3D cell culture systems provide a novel platform for investigating the cytoskeleton and intercellular microstress.

VEGF, an angiogenesis-related factor, activates the chondrocyte–endothelial crosstalk through the VEGF–NOTCH1–DLL4 pathway (Liu et al., 2025; Wang et al., 2025b). VEGF levels are affected by HIF-1α levels, which are regulated by the hypoxic microenvironment (Li et al., 2019; Tirpe et al., 2019). Moreover, abnormal stress accelerates angiogenesis and vascular invasion in the subchondral bone, and the process is triggered by PIEZO1 (Jiang et al., 2023; Xiao et al., 2025). As the chondrocyte–endothelial crosstalk progresses, the cartilage microenvironment collapses, further accelerating cartilage degeneration (Wang et al., 2025b). This important phenomenon was simulated in organoids using two kinds of cells, a feat impossible to replicate in simple *in vitro* cell cultures; notably, previous studies on this topic were primarily conducted *in vivo*. In this study, we used the endothelial cell line HMEC-1 to build an endothelial shell around the chondrocyte region in a 3D culture system, creating multi-cell-component organoids. Under inflammatory conditions, the endothelial cells invaded the chondrocyte core, mimicking the angiogenesis and vessel invasion characteristic of the subchondral bone and cartilage. Moreover, the VEGF–NOTCH1-DLL4 pathway may have activated the chondrocyte–endothelial crosstalk. Overall, use of the multi-cell-component organoids built using SW1353 and HMEC-1 cells is a feasible strategy for *in vitro* modeling of subchondral bone angiogenesis and chondrocyte–endothelial crosstalk. This strategy could overcome the limitations of simple *in vivo* studies.

Cartilage organoids constructed from stem cells, including ESCs, iPSCs, MSCs, and human periosteum-derived cells (hPDCs), have extensive applications in tissue engineering (Lin et al., 2023). However, highly demanding culture conditions and unpredictable cell differentiation pathways limit the application of cartilage organoids in disease modeling and drug screening. Although our tumor-derived organoids are unsuitable for regenerative medicine. Their simple culture method and controllable growth and differentiation make them valuable tools for disease modeling and drug screening. Moreover, our use of low-adhesion CSwell 600 grid microchambers provides a novel method for cartilage organoid engineering. These organoids can be differentiated into primary or alternative cell types, significantly broadening their application in research.

This study has few limitations. We analyzed HIF-1α levels to detect the hypoxic microenvironment, PIEZO1 levels to assess intercellular microstress, and VEGF–NOTCH1–DLL4 levels to examine chondrocyte–endothelial crosstalk. Although these markers are representative, they are not comprehensive. Besides, PIEZO1 is still and ion channel, and previous study have found that chondrocyte organoids still can simulate the intercellular effects mediated by ion channel, which we did not attach enough importance in this research (Liu et al., 2024). The study scheme needs to be modified as further research and application progress. Secondly, our current studies used SW1353 and HMEC-1 cells, which are both derived from tumor or immortalized cell lines. They may exhibit certain transformed phenotypes. It could be more reliable that test the organoids with OA drugs. However, there is no widely recognized and reliable drugs to cure OA, which means it meaningless to conduct positive control studies in current stage. Meanwhile, MIA could cause damage to both chondrocytes and endothelial cells, which could one of the potential factors for endothelial shell. Though, the inflammation stimulation and abnormal stress could cause simultaneous damage to both chondrocytes in cartilage and vessel endothelium in subchondral bone (Lin et al., 2025), there still need more study to discover in organoids *in vitro*. As results, this strategy could be only applied for phenotypic simulation. The further application needs to be explored in future. Thirdly, we only use male rats in this study, whether the effects have gender differences was need to be further explored.

**FIGURE 6:**
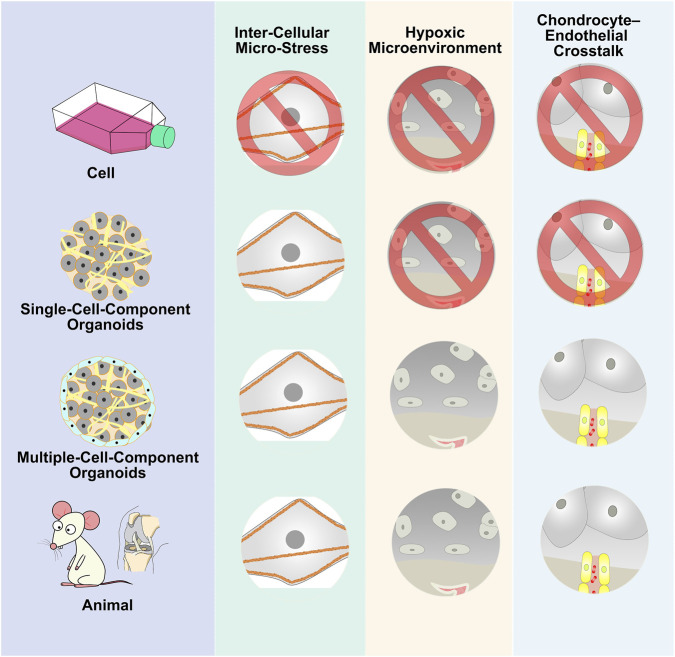
The main findings of this study. The multi-cell-component cartilage organoids partially simulated the intercellular microstress, hypoxic microenvironment, and chondrocyte–endothelial crosstalk observed in cartilage *in vivo*.

In conclusion as shown in Figure 6, the multi-cell-component cartilage organoids constructed from tumor-derived cell lines using low-adhesion grid microchambers partially simulated the intercellular microstress, hypoxic microenvironment, and chondrocyte–endothelial crosstalk observed in cartilage *in vivo*. These organoids offer a new strategy for conducting cartilage-related drug screening studies.

## Data Availability

The raw data supporting the conclusions of this article will be made available by the authors, without undue reservation.

## References

[B1] Abe K. Yamashita A. Morioka M. Horike N. Takei Y. Koyamatsu S. (2023). Engraftment of allogeneic iPS cell-derived cartilage organoid in a primate model of articular cartilage defect. Nat. Communications 14, 804. 10.1038/s41467-023-36408-0 36808132 PMC9941131

[B2] Abraham D. M. Herman C. Witek L. Cronstein B. N. Flores R. L. Coelho P. G. (2022). Self-assembling human skeletal organoids for disease modeling and drug testing. J. Biomedical Materials Research Part B, Appl. Biomaterials 110, 871–884. 10.1002/jbm.b.34968 34837719 PMC8854332

[B3] Brylka L. J. Alimy A. R. Tschaffon-Müller M. E. A. Jiang S. Ballhause T. M. Baranowsky A. (2024). Piezo1 expression in chondrocytes controls endochondral ossification and osteoarthritis development. Bone Research 12, 12. 10.1038/s41413-024-00315-x 38395992 PMC10891122

[B4] Garcia-Castorena J. M. Riester R. Gamino-Ornelas M. Ada N. Guilak F. Danalache M. (2025). PIEZO1-mediated calcium influx transiently alters nuclear mechanical properties *via* actin remodeling in chondrocytes. Biochem. Biophysical Research Communications 742, 151135. 10.1016/j.bbrc.2024.151135 39667069 PMC13273603

[B5] Gonzalez-Nolde S. Schweiger C. J. Davis E. E. R. Manzoni T. J. Hussein S. M. I. Schmidt T. A. (2024). The actin cytoskeleton as a regulator of proteoglycan 4. Cartilage 6, 19476035231223455. 10.1177/19476035231223455 38183234 PMC11569590

[B6] Hu S. Zhang C. Ni L. Huang C. Chen D. Shi K. (2020). Stabilization of HIF-1α alleviates osteoarthritis *via* enhancing mitophagy. Cell Death and Disease 11, 481. 10.1038/s41419-020-2680-0 32587244 PMC7316774

[B7] Jiang X. Li X. Fei X. Shen J. Chen J. Guo M. (2021). Endometrial membrane organoids from human embryonic stem cell combined with the 3D matrigel for endometrium regeneration in asherman syndrome. Bioact. Materials 6, 3935–3946. 10.1016/j.bioactmat.2021.04.006 33937593 PMC8079828

[B8] Jiang M. Zhang Y. X. Bu W. J. Li P. Chen J. H. Cao M. (2023). Piezo1 channel activation stimulates ATP production through enhancing mitochondrial respiration and glycolysis in vascular endothelial cells. Br. Journal Pharmacology 180, 1862–1877. 10.1111/bph.16050 36740831

[B9] Li Y. Sun R. Zou J. Ying Y. Luo Z. (2019). Dual roles of the AMP-activated protein kinase pathway in angiogenesis. Cells 8, 8. 10.3390/cells8070752 31331111 PMC6678403

[B10] Li M. Zhang X. Wang M. Wang Y. Qian J. Xing X. (2022). Activation of Piezo1 contributes to matrix stiffness-induced angiogenesis in hepatocellular carcinoma. Cancer Commun. Lond. Engl. 42, 1162–1184. 10.1002/cac2.12364 36181398 PMC9648387

[B11] Lin W. Wang M. Xu L. Tortorella M. Li G. (2023). Cartilage organoids for cartilage development and cartilage-associated disease modeling. Front. Cell Developmental Biology 11, 1125405. 10.3389/fcell.2023.1125405 36824369 PMC9941961

[B12] Lin K. Fan S. Yang X. Hou W. Zhang J. Liao J. (2025). Heterogeneity of active mast cells, endothelial cells, and fibroblasts in hemophilic arthritis defined by synovial single-cell sequencing. Sci. Reports 15, 43350. 10.1038/s41598-025-27368-0 41361221 PMC12686410

[B13] Liu Z. Zhou H. Wu Q. Luo T. Tu H. Sa G. (2024). Constructing condylar cartilage organoid to explore primary cilia functions. Heliyon 15 (10), e35972. 10.1016/j.heliyon.2024.e35972 39281559 PMC11395755

[B14] Liu Y. Da W. Xu M. J. Xiao C. X. Deng T. Zhou S. L. (2025). Single-cell transcriptomics reveals novel chondrocyte and osteoblast subtypes and their role in knee osteoarthritis pathogenesis. Signal Transduction Targeted Therapy 10, 40. 10.1038/s41392-025-02136-8 39904988 PMC11794573

[B15] Parreno J. Nabavi Niaki M. Andrejevic K. Jiang A. Wu P. H. Kandel R. A. (2017). Interplay between cytoskeletal polymerization and the chondrogenic phenotype in chondrocytes passaged in monolayer culture. J. Anatomy 230, 234–248. 10.1111/joa.12554 27807861 PMC5244456

[B16] Plaas A. Velasco J. Gorski D. J. Li J. Cole A. Christopherson K. (2011). The relationship between fibrogenic TGFβ1 signaling in the joint and cartilage degradation in post-injury osteoarthritis. Osteoarthr. Cartilage 19, 1081–1090. 10.1016/j.joca.2011.05.003 21624477

[B17] Qin H. J. Xu T. Wu H. T. Yao Z. L. Hou Y. L. Xie Y. H. (2019). SDF-1/CXCR4 axis coordinates crosstalk between subchondral bone and articular cartilage in osteoarthritis pathogenesis. Bone 125, 140–150. 10.1016/j.bone.2019.05.010 31108241

[B18] Rzepski A. T. Schofield M. M. Richardson-Solorzano S. Arranguez M. L. Su A. W. Parreno J. (2025). Targeting the reorganization of F-actin for cell-based implantation cartilage repair therapies. Differ. Research Biological Diversity 143, 100847. 10.1016/j.diff.2025.100847 40068531 PMC12162219

[B19] Sanz-Ramos P. Mora G. Vicente-Pascual M. Ochoa I. Alcaine C. Moreno R. (2013). Response of sheep chondrocytes to changes in substrate stiffness from 2 to 20 Pa: effect of cell passaging. Connect. Tissue Res. 54, 159–166. 10.3109/03008207.2012.762360 23323769

[B20] Taheem D. K. Jell G. Gentleman E. (2020). Hypoxia inducible Factor-1α in osteochondral tissue engineering. Tissue Engineering Part B, Rev. 26, 105–115. 10.1089/ten.TEB.2019.0283 31774026 PMC7166133

[B21] Tirpe A. A. Gulei D. Ciortea S. M. Crivii C. Berindan-Neagoe I. (2019). Hypoxia: overview on hypoxia-mediated mechanisms with a focus on the role of HIF genes. Int. Journal Mol. Sci. 5, 20. 10.3390/ijms20246140 31817513 PMC6941045

[B22] Wang Y. Liu G. Tao Z. Hua F. Zhao T. He H. (2025). PIEZO1-Mediated mechanotransduction in craniofacial biology. FASEB J. 31, e70937. 10.1096/fj.202501130R 40844383

[B23] Wang Z. Zhu P. Li H. Cheng J. Cai Y. (2025a). PDGF-BB inhibits F-actin formation and chondrocyte dedifferentiation in osteoarthritis *via* oxygen-dependent HIF-1α/SCIN regulation and RhoA/ROCK signaling inhibition. Eur. Journal Pharmacology 15, 178280. 10.1016/j.ejphar.2025.178280 41135736

[B24] Wang Z. Zhu P. Li H. Ye B. Luo Q. Cheng J. (2025b). Sodium Hyaluronate-PDGF repairs cartilage and subchondral bone microenvironment *via* HIF-1α-VEGF-Notch and SDF-1-CXCR4 inhibition in osteoarthritis. J. Cell. Mol. Med. 29, e70515. 10.1111/jcmm.70515 40159624 PMC11955409

[B25] Xiao B. (2024). Mechanisms of mechanotransduction and physiological roles of PIEZO channels. Nat. Rev. Mol. Cell Biol. 25, 886–903. 10.1038/s41580-024-00773-5 39251883

[B26] Xiao Y. Xu M. Shi Y. Wang J. Li Z. Xiao T. (2025). ZnCe-LDO nanozyme-based multifunctional hydrogel promotes bone regeneration by inflammatory macrophage reprogramming and Piezo1 activation. ACS Nano 5, 32606–32628. 10.1021/acsnano.5c10123 40911285 PMC12445009

[B27] Zeng D. Chen Y. Liao Z. Wei G. Huang X. Liang R. (2023). Cartilage organoids and osteoarthritis research: a narrative review. Front. Bioengineering Biotechnology 11, 1278692. 10.3389/fbioe.2023.1278692 38026876 PMC10666186

[B28] Zhang H. Wang L. Cui J. Wang S. Han Y. Shao H. (2023). Maintaining hypoxia environment of subchondral bone alleviates osteoarthritis progression. Sci. Advances 9, eabo7868. 10.1126/sciadv.abo7868 37018403 PMC10075992

[B29] Zhu W. J. Chang B. Y. Wang X. F. Zang Y. F. Zheng Z. X. Zhao H. J. (2020). FBW7 regulates HIF-1α/VEGF pathway in the IL-1β induced chondrocytes degeneration. Eur. Review Medical Pharmacological Sciences 24, 5914–5924. 10.26355/eurrev_202006_21484 32572904

